# Lack of Association between Serum Interleukin-23 and Interleukin-27 Levels and Disease Activity in Patients with Active Systemic Lupus Erythematosus

**DOI:** 10.3390/jcm10204788

**Published:** 2021-10-19

**Authors:** Katarzyna Pawlak-Buś, Wiktor Schmidt, Piotr Leszczyński

**Affiliations:** 1 Department of Rheumatology, Rehabilitation and Internal Medicine, Poznań University of Medical Sciences, 61-701 Poznań, Poland; wiktorpawelschmidt@gmail.com (W.S.); piotr_leszczynski@wp.pl (P.L.); 2 Department of Rheumatology and Osteoporosis, J. Struś Municipal Hospital in Poznań, 61-285 Poznań, Poland

**Keywords:** systemic lupus erythematosus, neuropsychiatric lupus, interleukin-23, interleukin-27, SLEDAI

## Abstract

Systemic lupus erythematosus (SLE) is a chronic systemic autoimmune disease characterized by the production of multiple autoantibodies, resulting in tissue and organ damage. Recent studies have revealed that interleukin-23 (IL-23) and interleukin-27 (IL-27) may be therapeutically relevant in selected SLE manifestations. This study aimed to identify associations between serum IL-27 and IL-23 levels and disease activity in Polish patients with different manifestations of SLE: neuropsychiatric lupus (NPSLE), and lupus nephritis (LN). Associations between interleukin levels and oligo-specific antibodies against double-stranded DNA (dsDNA), dose of glucocorticoids, and type of treatment were also analyzed. An enzyme-linked immunosorbent assay was used to assess anti-dsDNA antibodies and analyze the serum concentration of IL-27 and IL-23 from 72 patients aged 19–74 years with confirmed active SLE. Disease activity was measured using the Systemic Lupus Erythematosus Disease Activity Index 2000 (SLEDAI 2-K). No significant correlations between interleukin levels and SLEDAI score, anti-dsDNA, corticosteroid dose, or type of treatment were noted. Patients with NPSLE and LN presented the highest median scores of SLEDAI.

## 1. Introduction

Systemic lupus erythematosus (SLE) is a complex autoimmune disease with unclear pathogenesis that causes systemic inflammation [1]. Dysfunction of the immune system involves the production of multiple autoantibodies and the formation and deposition of immune complexes. This contributes to damage to various organs [2], including the kidneys and both the central and peripheral nervous systems [3]. Consequently, two common SLE manifestations are neuropsychiatric lupus (NPSLE) and lupus nephritis (LN). Lupus nephritis, observed in ~30% of SLE patients [4], is the primary SLE complication [5]. The prevalence of NPSLE varies widely, as it is estimated that it may affect between 37% and 95% of SLE patients [6]. Up to 75% of adult and pediatric patients with SLE will experience various disabling effects of NPSLE that impact their quality of life and prognosis [7]. In Poland, the population of treated SLE patients is highly stable, at ~20,000 per year [8].

In recent years, an increasing number of authors have analyzed the role of cytokines in the pathogenesis of SLE [9,10,11,12,13,14]. It has been shown that crucial features of SLE’s pathogenesis and progression include aberrations in the T-lymphocyte compartment and abnormal cytokine production [15]. Interleukin-23 (IL-23) and interleukin-27 (IL-27) regulate T helper 1 (Th1)-cell responses. IL-23 plays an important role in autoimmune inflammation by stimulating a unique T-cell subset to produce interleukin-17 (IL-17) [16,17]. In contrast, IL-27 is responsible for the reduction in the intensity and duration of adaptive immune responses [18,19]. Some studies have shown that IL-27 controls the development of T helper 17 (Th17) cells, which are implicated in the pathogenesis of SLE [20]. Moreover, the elevated gene expression of IL-27 was found in immune cells from SLE patients with a high type I interferon (I-IFN) signature, which further confirms the importance of IL-27 in SLE [21].

The role of both interleukins in the pathogenesis of SLE, their association with disease activity, and their therapeutic potential have been analyzed over the past decade; however, these studies were based on small sample sizes, and provided inconsistent results [20,22,23,24,25,26,27,28]. In patients with SLE, it was shown that serum IL-23 concentration is higher [24,25,26,27] and IL-27 concentration is lower when compared to healthy controls [20,23]. Most authors found a positive correlation between IL-23 concentration and disease activity [24,28], and a lack of correlation between IL-27 levels and disease activity [20]. Higher levels of IL-23 were also associated with LN [24,25,27]. Vukelic et al. found that increased IL-23 levels are characteristic of patients with LN, but also patients with non-renal lupus. Furthermore, they observed the correlation between increased levels of IL-23 and positive anti-dsDNA antibodies and/or low C3 levels [28]. Interestingly, Xia et al. showed that both IL-23 and IL-27 urine levels were significantly correlated with the renal SLE Disease Activity Index (SLEDAI) [26]. On the other hand, Li et al. and Gaber et al. reported a negative association of IL-27 levels and the occurrence of LN [20,23]. These results suggest that the concentration of both interleukins may be more significantly associated with disease activity in patients with LN. To the best of our knowledge, there are no similar analyses for patients with NPSLE.

The primary aim of this study was to identify associations between serum IL-27 and IL-23 levels and disease activity in Polish patients with different manifestations of SLE (LN and NPSLE). We also assessed the association between interleukin levels and oligo-specific antibodies against double-stranded DNA (dsDNA), the dose of glucocorticoids (GCs), and the type of treatment in the analyzed groups of patients.

## 2. Materials and Methods

### 2.1. Diagnostic Criteria

Patients were considered eligible for the study if they fulfilled the criteria of the American College of Rheumatology (ACR) classification for SLE [29], and if they were diagnosed as having clinically active SLE qualifying for treatment. According to the ACR and the European League Against Rheumatism (EULAR) [30], one of the criteria of SLE is positive antinuclear antibodies (ANAs) at a titer of 1:80 or greater [31]. Another biomarker of SLE is the presence of oligo-specific antibodies against dsDNA at a nominal value of 100 units/ampoule (WHO Reference Reagent for lupus) [32].

The diagnostics included the profile of autoantibodies determined via indirect immunofluorescence assay (IIFA) and sandwich enzyme-linked immunosorbent assay (ELISA).

Lupus nephritis was confirmed via kidney involvement (proteinuria, active urine sediment) and/or kidney biopsy; NPSLE was confirmed via neuropsychiatric manifestations under the 1999 ACR nomenclature [33].

### 2.2. Methodology

This is a retrospective, cross-sectional study. Clinical information was assessed with the application of a questionnaire that included:

- Demographic data (age, sex);
- Medical history/clinical data;
- Current treatment;
- Laboratory results (titer and profile of ANAs [31], anti-dsDNA [34], complement components C3 and C4 concentrations, and serum IL-23 and IL-27 concentrations);
- Measurements (morphology, biochemistry, urinalysis, daily proteinuria);
- Disease activity measured with the Systemic Lupus Erythematosus Disease Activity Index 2000 (SLEDAI 2-K) [35,36], Physician Global Assessment (PGA) [37], and organ damage determined using the Systemic Lupus International Collaborating Clinics/American College of Rheumatology (SLICC/ACR) Damage Index (SDI) [38].

The SLEDAI is a global index for the assessment of lupus disease activity in the previous 10 days; it consists of 24 weighted clinical and laboratory variables of 9 organ systems. The scores of the descriptors range from 1 to 8, and the total possible score for all 24 descriptors is 105 [36].

The PGA is a visual analogue score, recommended in the recent European League Against Rheumatism (EULAR) guidelines [39], for evaluating disease activity, treatment response, and remission in SLE [37]. 

The SLICC/ACR DI was used to measure damage—defined as irreversible organ dysfunction, present for 6 months or longer, regardless of etiology—in all organ systems. The SLICC/ACR DI was calculated based on organ damage accumulated since the onset of SLE up until the last visit [38].

### 2.3. Immunoassays

Blood samples were collected from patients during their admission to the hospital due to SLE activity, and immediately frozen at a temperature below 70 °C, then thawed and measured without repeated freeze/thawing. All of these activities were performed under standardized conditions to enable direct comparison of the results.

IgG ANAs were assessed in the HEp-2 cell line using the IIFA technique. Anti-dsDNA antibodies were assessed via monospecific sandwich ELISA tests. Concentrations of serum interleukins were measured via ELISA, using the Human IL-23 Quantikine ELISA Kit (R&D Systems) and Invitrogen™ eBioscience™ Human IL-27 Platinum ELISA Kit according to their respective manuals. The reaction results were measured using an EPOCH BioTek plate reader spectrophotometer at 450 nm, and calculated as pg/mL.

### 2.4. Statistics

Data distribution was checked using the Shapiro–Wilk test. Data with normal distribution were presented as mean ± SD, and non-parametrical data were presented as median and range. Differences in selected parameters between two groups of patients were assessed using Student’s *t*-test or the Mann–Whitney test. One-way analysis of variance or the Kruskal–Wallis test were used to determine whether there were significant differences in the analyzed parameters between patients with different SLE manifestations. Spearman’s correlation was applied to assess associations between measures. Logistic regression was used to calculate the odds ratios (ORs) and 95% confidence intervals (CIs) for selected determinants concerning disease activity in the whole group of patients, and in selected groups of patients with LN and NPSLE. STATISTICA 12 (StatSoft Inc., Tulsa, OK, USA) and R 4.0.2 (R Statistics) software were used.

### 2.5. Ethics

This study obtained approval from the Bioethical Committee of the Poznań University of Medical Sciences (no. 107/21).

## 3. Results

### 3.1. Patient Characteristics

A total of 144 Caucasian patients from the Department of Rheumatology and Osteoporosis, Józef Struś Specialist Municipal Hospital, Poznań, Poland were screened. Results of 72 patients aged 19–74 years with confirmed active SLE (NPSLE—29%; LN—22%; NPSLE + LN—7%; non-LN and non-NPSLE—42%) were included in the statistical analysis. Details concerning clinical characteristics are presented in Table 1. Patients were treated with chloroquine (CQ, *n* = 47, 65%), hydroxychloroquine (HCQ, *n* = 13, 18%), glucocorticoids (GCs, *n* = 59, 82%) (<7.5 mg, *n* = 28, 7.5–10 mg, *n* = 13, >10 mg, *n* = 16), and the following immunosuppressant medications (IS, *n* = 58, 81%): cyclophosphamide (CTX, *n* = 10, 14%), mycophenolate mofetil (MMF, *n* = 16, 22%), methotrexate (MTX, *n* = 6, 8%), azathioprine (AZA, *n* = 21, 29%), and cyclosporine A (CsA, *n* = 5, 7%). Among 21 patients with LN (16 with LN, 5 with LN and NPSLE), 7 had renal biopsy (*n* = 3, class III nephritis; and *n* = 4, class IV nephritis). Antiphospholipid syndrome was confirmed in 6 (8%) patients, whereas antiphospholipid antibodies were confirmed in 11 (15%).

### 3.2. Disease Activity and Laboratory Test Results

Table 2 presents the analysis of variance for disease assessment, laboratory results, and interleukin levels in the whole study group and in the NPSLE/LN subgroups. There were significant differences in SLEDAI and PGA scores between patients with different manifestations, i.e., patients with NPSLE and LN presented the highest median scores of SLEDAI and PGA. There were no significant differences in the other measured variables. 

### 3.3. Determinants of Disease Activity and Different Disease Manifestations

The independent variables that were associated with SLEDAI scores in the whole group of patients were age at disease onset, decreased C3/C4, and anti-dsDNA (Table 3). 

A total of 24 patients (NPSLE = 7; LN = 7; NPSLE and LN = 1; non-LN and non-NPSLE = 9) presented cutaneous and musculoskeletal manifestations. The SLEDAI score (median, range) in this group was 17.5 (8.0–36.0), and in selected subgroups, was as follows: NPSLE = 26.0 (17.0–36.0); LN = 16.0 (12.0–27.0); non-LN and non-NPSLE = 12.0 (8.0–20.0).

There were no significant independent variables associated with the presence of LN (Table 4). Age at disease onset was the only significant determinant associated with the presence of NPSLE (Table 5).

### 3.4. Association between IL-23/IL-27 Levels and Disease Activity

**Figure 1:**
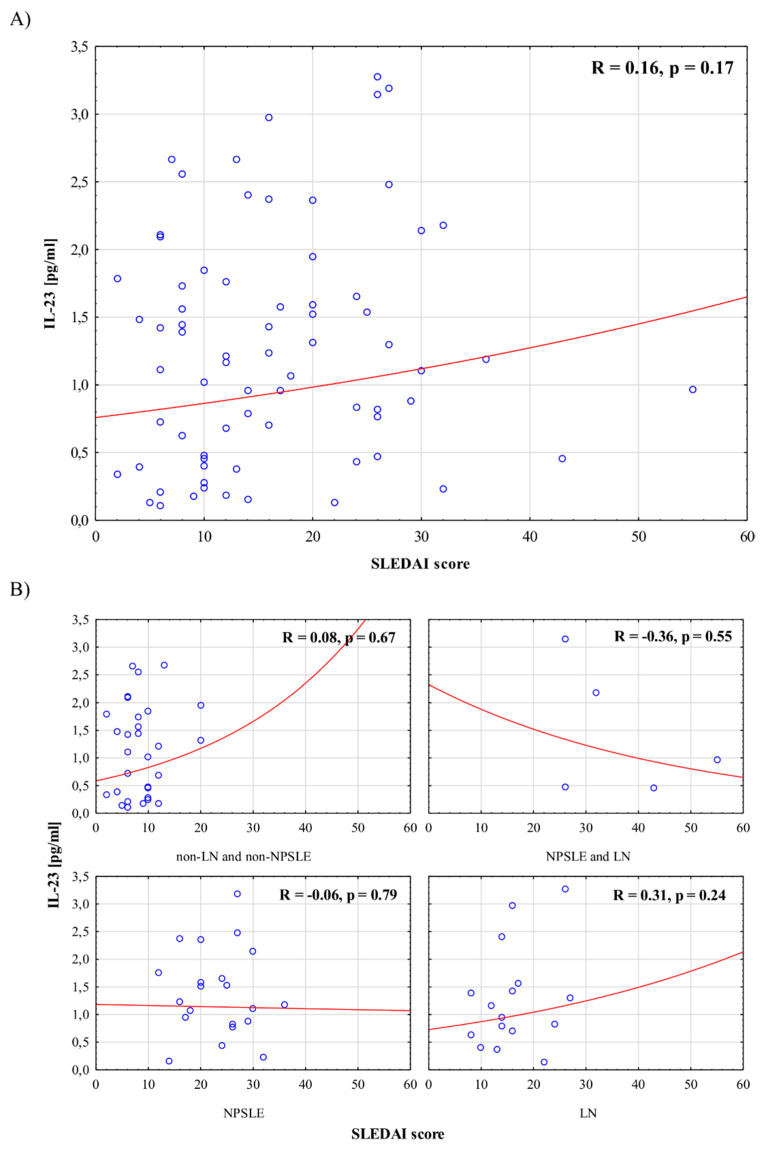
Association between serum IL-23 levels and disease activity in the whole group of patients (**A**), and in selected groups of patients (**B**).

**Figure 2:**
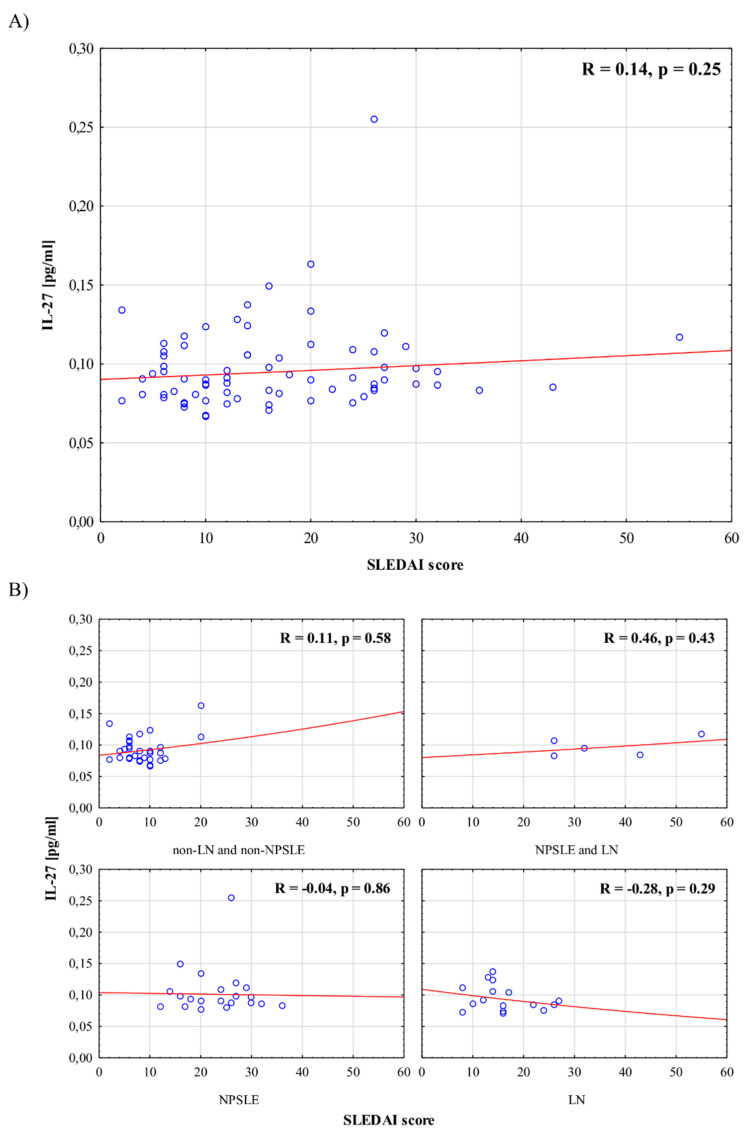
Association between serum IL-27 levels and disease activity in the whole group of patients (**A**), and in selected groups of patients (**B**).

Figure 1 and Figure 2 present associations between serum IL-23 and IL-27 levels, respectively, and disease activity in the whole group of patients, as well as in selected subgroups with different SLE manifestations. There were no significant correlations between interleukin levels and SLEDAI scores in the whole group, nor in the subgroups. 

To employ multiple tools of disease activity measurement, we checked the associations between serum IL-23 and IL-27 levels and SLEDAI scores in patients with PGA > 0. We found no correlation—R was 0.04 (*p* = 0.79) for IL-23, and R was 0.13 (*p* = 0.352) for IL-27. 

Furthermore, there was no significant correlation between interleukin levels and anti-dsDNA (Supplementary Materials, Figure S1).

### 3.5. Associatiosn between IL-23 Levels, IL-27 Levels, dsDNA, and Complement C3/C4 Components

There were no associations between elevated anti-dsDNA and low C3/C4, IL-23, or IL-27 (Table 6).

### 3.6. Association between IL-23/IL-27 Levels and SLE Treatment

There was no significant correlation between interleukin levels and corticosteroid dose (Supplementary Materials, Figure S2), or between interleukin levels and type of treatment (Supplementary Materials, Figure S3). 

## 4. Discussion

In the present study, we analyzed associations between IL-23 and IL-27 levels and disease activity in patients with SLE. We decided to stratify patients according to renal and neurological involvement in order to investigate IL-23 and IL-27 in the patients with the highest SLE activity in the respective organs, thereby identifying the highest risk of damage. We found a lack of correlation between serum IL-23 and IL-27 levels and disease activity measured with the SLEDAI in both the whole group and the selected subgroups of patients with different manifestations of the disease. Nevertheless, one can notice that patients with NPSLE and LN presented the highest SLEDAI and PGA scores. Nominally, the highest median value of IL-23 concentration was observed in patients with NPSLE. Additionally, we found that in the whole group of patients, there was no significant association between interleukin levels and anti-dsDNA, dose of GCs, or type of treatment. Moreover, there were no significant differences in interleukin levels between patients with and without immunologically active disease. 

In recent years, an increasing number of studies have been published analyzing the possible role of IL-23 and IL-27 (members of the IL-12 family) in the pathogenesis of SLE, and their potential contribution to immune imbalance [9,20,21,22,23,24,25,26,27,28]. Differences in both IL-23 and IL-27 levels between patients with SLE and healthy controls were shown. Several authors showed that the IL-27 levels were significantly lower, whereas the IL-23 levels were significantly higher, in SLE patients in comparison to healthy controls [20,23,24,25,27]. Contrary to our results, Hegab et al. [24] and, more recently, Vukelic et al. [28] found a positive correlation between IL-23 levels and disease activity measured with the SLEDAI. On the other hand, our results are consistent with data presented by Li et al. [20], showing no correlation between IL-27 levels and disease activity. 

We also evaluated interleukin levels in patients with various manifestations of SLE. We found that, in patients with LN and NPSLE + LN, IL-23 levels were nominally (but not significantly) lower, while IL-27 levels were similar to other subgroups of patients with different manifestations of SLE. However, there was no association between interleukin levels and disease activity in these subgroups (LN subgroup and NPSLE + LN subgroup). Several authors found high levels of IL-23 in patients with LN in comparison to healthy controls [24,25,27], and a negative association between IL-27 levels and LN occurrence [20,23]. In other research, IL-23 and IL-27 levels were positively and negatively associated with the renal SLEDAI, respectively [26]. To the best of our knowledge, there have been no studies on the association between IL-23 and/or IL-27 levels and disease activity in patients with NPSLE.

In our study, an association between interleukin levels and anti-dsDNA antibodies was not confirmed for IL-23 or IL-27. However, interleukin levels in immunologically active patients were nominally higher. Immunologically active patients and patients with high SLEDAI were treated with immunosuppressants and GCs, which could have a significant impact on the pro-inflammatory effect of IL-23. In 2020, Vukelic et al. showed a strong correlation between IL-23 levels and anti-dsDNA antibodies [28]. In an animal model of lupus, deletion of the IL-23 receptor blocks the interleukin signaling [40] and, consequently, decreases the production of anti-dsDNA antibodies [28]. However, there are some differences between our study and the Vukelic et al. study. Our group consisted of Caucasian patients who presented a median SLEDAI value of 14, whereas in Vukelic et al.’s study, the mean SLEDAI value in patients of different ethnicities was 6.7 [28].

In the present study, GC doses were not associated with the levels of either interleukin in patients with active SLE, i.e., patients with higher doses of medications did not present lower values of IL-23 or IL-27. These results are, to some extent, consistent with those presented in 2020 by Vukelic et al., who showed that immunomodulatory medications used for mild or severe LN did not affect IL-23 levels [28]; the authors suggested that drugs commonly used in patients with SLE may not be effective in shutting down the IL-23/IL-17 axis [28]. However, some authors found that six months of immunosuppressive treatment (GCs together with CTX or MMF) may decrease urine IL-23 concentrations in patients with a complete response, supporting the potential role of IL-23 in the pathogenesis of LN [26]. 

In our study, patients with NPSLE presented the highest serum concentrations of IL-23. This group of patients may be predisposed to IL-23 blockade treatment response [22]. IL-23 has pro-inflammatory and inhibitory functions; it is produced in response to microbial pathogens, and is essential for the differentiation of naïve CD4 T cells [41]. Anti-IL-23 therapy is intended to inhibit multiple inflammatory pathways critical for driving autoimmune inflammation [24,42,43]. Recent data suggest that IL-23 inhibitors offer safe and effective treatment of autoimmune inflammatory diseases. Ustekinumab, the first agent of this pharmacological class—also targeting IL-12/IL-23p40—has been approved for Crohn’s disease, ulcerative colitis, plaque psoriasis, and psoriatic arthritis treatment. However, despite the initially promising phase II trial results, Janssen recently announced the discontinuation of the phase III LOTUS study evaluating ustekinumab (STELARA^®^) in patients with SLE, due to lack of efficacy [44].

Risankizumab has been approved for the treatment of moderate-to-severe plaque psoriasis and, in Japan, also for psoriasis vulgaris, generalized pustular psoriasis, erythrodermic psoriasis, and psoriatic arthritis [41,45,46,47,48,49,50].

Another IL-23 blocker, guselkumab, approved for the treatment of plaque psoriasis and psoriatic arthritis, also has the potential to treat patients with Crohn’s disease [47,50,51,52,53,54] and ulcerative colitis [50,55,56,57].

Some reports suggest that patients with positive anti-dsDNA antibodies and/or low C3 levels are more likely to have elevated levels of IL-23 [28] and, due to immunological disease activity and lack of full disease remission, are in a high-risk group for “immune-mediated” and “treatment-mediated” tissue damage. In our study, patients with positive anti-dsDNA antibodies and low C3/C4 presented higher serum concentrations of IL-23 than those with negative anti-dsDNA antibodies; however, the difference was not statistically significant. Patients with clinically and immunologically active SLE require more aggressive therapy, which significantly modifies cytokine activity [58].

An increase in IL-27 serum concentration is a protective response to pathogenic factors, preventing the impairment of tissues and organs due to the promotion of specific Treg cell subsets, and inhibition of Th1, Th2, Th17, and antigen-presenting cells [59]. In our study, levels of both interleukins were not related to the type of treatment in the whole group of patients. Xia et al. showed that IL-27 levels can be successfully increased after six months of treatment with GCs together with CTX or MMF in patients with LN [26]. The discrepancies between the abovementioned studies may result from differences in the immunological and clinical activity of SLE, the degree of the therapy aggressiveness, the heterogeneity of the disease, and small sample sizes. 

Due to scarce and inconsistent data that hinder the drawing of unambiguous conclusions, the exact roles of IL-27 and IL-23 in the pathogenesis of SLE should be verified in future studies.

The heterogeneous group of patients, lack of a control group, and the retrospective nature of our work should be mentioned here as limitations of our study.

## 5. Conclusions

Our findings suggest a lack of correlation between serum IL-23 and IL-27 levels and disease activity measured with the SLEDAI in a group of patients with different manifestations of SLE disease. We additionally found no significant associations between interleukin levels and anti-dsDNA, dose of GCs, or type of treatment. Moreover, no significant differences in interleukin levels between patients with and without immunologically active disease were observed.

## Figures and Tables

**Table 1:** Clinical characteristics of the study group and neuropsychiatric lupus/lupus nephritis subgroups.

Variables	All Patients	Non-LN and Non-NPSLE	NPSLE	LN	NPSLE + LN	*p*
*n*	72	30	21	16	5	-
Sex (♂/♀)	4/68	2/28	2/19	0/16	0/5	0.39
Age (mean ± SD) (years)	42.9 ± 13.3	47.7 ± 12.2	38.3 ± 11.4	42.1 ± 14.6	36.4 ± 16.9	<0.05
Age at disease onset (mean ± SD) (years)	36.5 ± 12.6	41.9 ± 10.8	32.5 ± 10.7	33.6 ± 14.4	29.4 ± 14.8	<0.05
Disease duration (median, min–max) (years)	5, 1–20	5, 1–12	4, 1–15	5, 1–20	7, 1–12	0.78
Fever (yes/no)	4/68	0/30	1/20	2/14	1/4	0.12
Lupus rash (yes/no)	53/19	22/8	5/16	4/12	2/3	0.91
Alopecia (yes/no)	45/27	19/11	12/9	12/4	2/3	0.49
Mucosal ulcers (yes/no)	5/67	0/30	2/19	2/14	1/4	0.11
Arthritis (yes/no)	53/19	21/9	15/6	13/3	4/1	0.83
Myositis (yes/no)	0/72	0/30	0/21	0/16	0/5	-
Psychosis (yes/no)	4/68	0/30	4/17	0/16	5/0	<0.05
Organic brain syndrome (yes/no)	25/47	0/30	21/0	0/16	4/1	<0.001
Cranial nerves disorder (yes/no)	1/71	0/30	1/20	0/16	0/5	0.48
Vision disturbances (yes/no)	1/71	0/30	1/20	0/16	0/5	0.48
Lupus headache (yes/no)	6/66	0/30	5/16	0/16	1/4	<0.01
Cerebrovascular accident (yes/no)	2/70	0/30	1/20	0/16	1/4	0.16
Vasculitis (yes/no)	11/61	2/28	4/17	3/13	2/3	0.24
Pleuritis (yes/no)	4/68	0/30	2/19	0/16	2/3	<0.05
Pericarditis (yes/no)	2/70	0/30	0/21	1/15	1/4	0.12
Active urinary sediment (yes/no)	3/69	0/30	0/21	1/15	2/3	<0.05
Hematuria (yes/no)	2/70	0/30	0/21	0/16	2/3	<0.01
Proteinuria (yes/no)	18/54	0/30	0/21	13/3	5/0	<0.001
Leukocyturia (yes/no)	7/65	0/30	0/21	5/11	2/3	<0.001
Leukopenia (yes/no)	11/61	4/26	5/16	1/15	1/4	0.48
Thrombocytopenia (yes/no)	9/63	4/26	1/20	2/14	2/3	0.28

NPSLE: neuropsychiatric lupus; LN: lupus nephritis.

**Table 2:** Disease assessment, laboratory test results, and interleukin levels in the study group and neuropsychiatric lupus/lupus nephritis subgroups.

Variables	All Patients	Non-LN and Non-NPSLE	NPSLE	LN	NPSLE + LN	*p*
SLEDAI (median, min–max) (points)	14, 2–55	8, 2–20	24, 12–36	15, 8–27	32, 26–55	<0.001
PGA (median, min–max) (points)	1, 0–3	1, 0–3	2, 0–3	1, 0–3	3, 0–3	<0.05
SDI (yes/no)	30/42	11/19	8/13	9/7	2/3	0.61
Low C3/C4 (yes/no)	36/36	13/17	11/10	11/5	1/4	0.19
Elevated anti-dsDNA (yes/no)	49/23	16/14	16/5	12/4	5/0	0.05
Elevated anti-dsDNA and low C3/C4 (yes/no)	33/39	11/19	10/11	10/6	2/3	0.41
IL-23 (median, min–max) (pg/mL)	1.18, 0.11–3.28	1.16, 0.11–2.67	1.23, 0.16–3.19	1.06, 0.14–3.28	0.96, 0.46–3.15	0.70
IL-27 (median, min–max) (pg/mL)	0.09, 0.07–0.26	0.09, 0.07–0.16	0.09, 0.08–0.26	0.09, 0.07–0.14	0.10, 0.08–0.12	0.47

NPSLE: neuropsychiatric lupus; LN: lupus nephritis; SLEDAI: Systemic Lupus Erythematosus Disease Activity Index; PGA: Physician Global Assessment; SDI: Systemic Lupus International Collaborating Clinics/American College of Rheumatology (SLICC/ACR) Damage Index; anti-dsDNA: autoantibodies against double-stranded DNA; IL: interleukin.

**Table 3:** Determinants of disease activity in the whole group of patients.

	SLEDAI Score (Points)			Odds Ratio		
Determinant	*n*	1–11, *n* = 27 *n* (%)	≥12, *n* = 45 *n* (%)	OR	95% CI	*p*
Disease duration (years)	72					0.33
[1, 4]		9 (33%)	23 (51%)	—	—	
(4, 7.57]		7 (26%)	9 (20%)	0.50	0.14, 1.78	
(7.57, 20]		11 (41%)	13 (29%)	0.46	0.15, 1.40	
Age at disease onset (years) median (IQR)	72	42 (39, 50)	35 (22, 43)	0.94	0.90, 0.98	<0.01
IL-27 (pg/mL)	72					0.29
[0.067, 0.0837]		12 (44%)	12 (27%)	—	—	
(0.0837, 0.0983]		7 (26%)	17 (38%)	2.43	0.75, 8.31	
(0.0983, 0.255]		8 (30%)	16 (36%)	2.00	0.63, 6.62	
IL-23 (pg/mL)	72					0.10
[0.112, 0.776]		13 (48%)	11 (24%)	—	—	
(0.776, 1.55]		6 (22%)	18 (40%)	3.55	1.08, 12.8	
(1.55, 3.28]		8 (30%)	16 (36%)	2.36	0.75, 7.87	
Decreased C3/C4	72					<0.01
No		19 (70%)	17 (38%)	—	—	
Yes		8 (30%)	28 (62%)	3.91	1.45, 11.4	
Anti-dsDNA	72					<0.001
No		15 (56%)	8 (18%)	—	—	
Yes		12 (44%)	37 (82%)	5.78	2.03, 17.8	

SLEDAI: Systemic Lupus Erythematosus Disease Activity Index; OR: odds ratio; CI: confidence interval; anti-dsDNA: autoantibodies against double-stranded DNA; IL: interleukin.

**Table 4:** Determinants of disease activity in a group of LN patients.

	LN			Odds Ratio		
Determinant	*n*	0, *n* = 51 *n* (%)	1, *n* = 21 *n* (%)	OR	95% CI	*p*
Disease duration (years)	72					0.84
[1, 4]		23 (45%)	9 (43%)	—	—	
(4, 7.57]		12 (24%)	4 (19%)	0.85	0.20, 3.23	
(7.57, 20]		16 (31%)	8 (38%)	1.28	0.40, 4.06	
Age at disease onset (years) median (IQR)	72	40 (31, 44)	28 (21, 45)	0.97	0.92, 1.01	0.09
IL-27 (pg/mL)	72					0.82
[0.067, 0.0837]		18 (35%)	6 (29%)	—	—	
(0.0837, 0.0983]		17 (33%)	7 (33%)	1.24	0.34, 4.56	
(0.0983, 0.255]		16 (31%)	8 (38%)	1.50	0.43, 5.46	
IL-23 (pg/mL)	72					0.82
[0.112, 0.776]		17 (33%)	7 (33%)	—	—	
(0.776, 1.55]		16 (31%)	8 (38%)	1.21	0.36, 4.22	
(1.55, 3.28]		18 (35%)	6 (29%)	0.81	0.22, 2.92	
Decreased C3/C4	72					0.44
No		27 (53%)	9 (43%)	—	—	
Yes		24 (47%)	12 (57%)	1.50	0.54, 4.27	
Anti-dsDNA	72					0.12
No		19 (37%)	4 (19%)	—	—	
Yes		32 (63%)	17 (81%)	2.52	0.79, 9.76	

OR: odds ratio; CI: confidence interval; anti-dsDNA: autoantibodies against double-stranded DNA; IL: interleukin, 0 = patients without LN, 1 = patients with LN.

**Table 5:** Determinants of disease activity in a group of NPSLE patients.

	NPSLE			Odds Ratio		
Determinant	*n*	0, *n* = 46 *n* (%)	1, *n* = 26 *n* (%)	OR	95% CI	*p*
Disease duration (years)	72					0.55
[1, 4]		19 (41%)	13 (50%)	—	—	
(4, 7.57]		12 (26%)	4 (15%)	0.49	0.12, 1.76	
(7.57, 20]		15 (33%)	9 (35%)	0.88	0.29, 2.59	
Age at disease onset (years) median (IQR)	72	42 (31, 48)	32 (22, 39)	0.95	0.91, 0.99	<0.05
IL-27 (pg/mL)	72					0.31
[0.067, 0.0837]		18 (39%)	6 (23%)	—	—	
(0.0837, 0.0983]		13 (28%)	11 (42%)	2.54	0.76, 9.09	
(0.0983, 0.255]		15 (33%)	9 (35%)	1.80	0.53, 6.49	
IL-23 (pg/mL)	72					0.37
[0.112, 0.776]		18 (39%)	6 (23%)	—	—	
(0.776, 1.55]		14 (30%)	10 (38%)	2.14	0.64, 7.69	
(1.55, 3.28]		14 (30%)	10 (38%)	2.14	0.64, 7.69	
Decreased C3/C4	72					0.62
No		22 (48%)	14 (54%)	—	—	
Yes		24 (52%)	12 (46%)	0.79	0.30, 2.06	
Anti-dsDNA	72					0.08
No		18 (39%)	5 (19%)	—	—	
Yes		28 (61%)	21 (81%)	2.70	0.91, 9.24	

OR: odds ratio, CI: confidence interval; 0 = patients without NPSLE, 1 = patients with NPSLE.

**Table 6:** Differences in IL-23 and IL-27 (median, min–max) between patients with and without elevated anti-dsDNA and decreased C3/C4.

	Elevated Anti-dsDNA and Low C3/C4 No (*n* = 39)	Elevated Anti-dsDNA and Low C3/C4 Yes (*n* = 33)	*p*
IL-23 (pg/mL)	0.96, 0.11–3.28	1.30, 0.14–3.19	0.31
IL-27 (pg/mL)	0.09, 0.07–0.16	0.09, 0.07–0.26	0.63

dsDNA: double-stranded DNA.

## Data Availability

Restrictions apply to the availability of these data. Data were obtained from J. Struś Municipal Hospital in Poznań, Poland, and are available from the corresponding author with the permission of the hospital authorities.

## Supplementary Materials

The following are available online at https://www.mdpi.com/article/10.3390/jcm10204788/s1: Figure S1: Association between serum IL-27 and IL-23 levels and anti-dsDNA; Figure S2: Association between corticosteroid dose and IL-23 and IL-27 levels. Corticosteroid doses expressed as prednisolone equivalents were classified as follows: 1: ≤7.5 mg; 2: 7.6–10 mg; 3: >10 mg; 4: high intravenous corticosteroid doses; Figure S3: Association between IL-23 and IL-27 levels and type of treatment. CS: corticosteroids; IS: immunosuppressant medications; whiskers: min–max, inside box corresponds to the median, outside box boundaries correspond to 25% and 75%.
