# Supplementary material for: Lack of Association between Serum Interleukin-23 and Interleukin-27 Levels and Disease Activity in Patients with Active Systemic Lupus Erythematosus
Source: J Clin Med. 2021 Oct 19;10(20):4788. doi: 10.3390/jcm10204788 (PMC8537777; doi:10.3390/jcm10204788)
Supplement: Supplementary file 1 [file jcm-10-04788-s001.zip › jcm-1383743-supplementary.pdf]

Association between serum interleukin-23 and interleukin-27 levels and disease activity in Polish patients with active systemic lupus erythematosus

Katarzyna Pawlak-Buś, Wiktor Schmidt, Piotr Leszczyński

Supplementary material

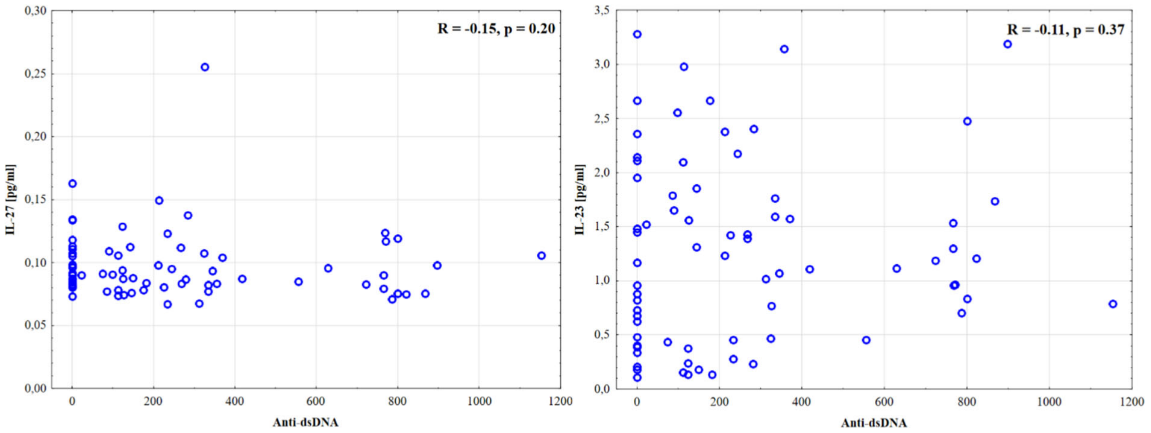

Figure S1. Association between serum IL-27 and IL-23 levels and anti-dsDNA.

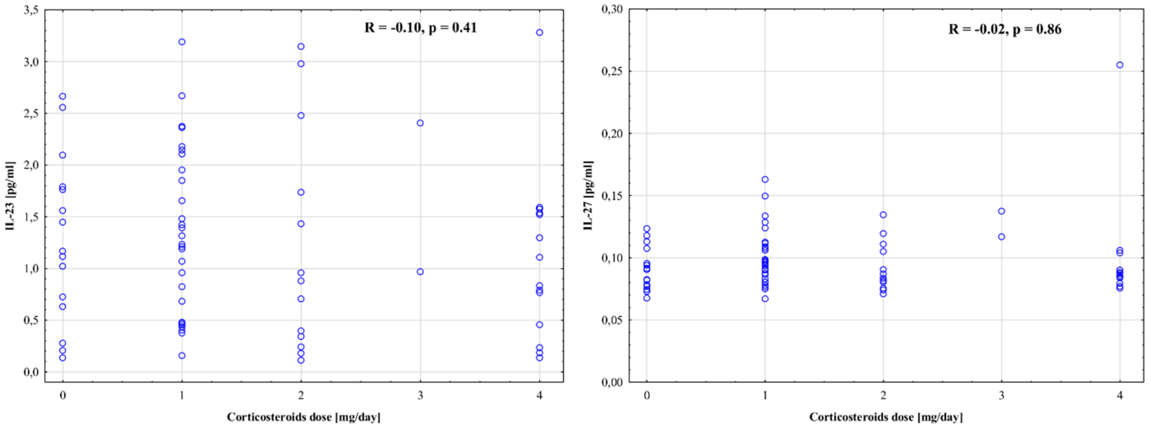

Figure S2. Association between corticosteroids dose and IL-23 and IL-27 levels. Corticosteroid doses expressed as prednisolone equivalency were classified as follows: 1: ≤ 7.5 mg; 2: 7.6–10 mg; 3: > 10 mg; 4: high intravenous corticosteroids doses.

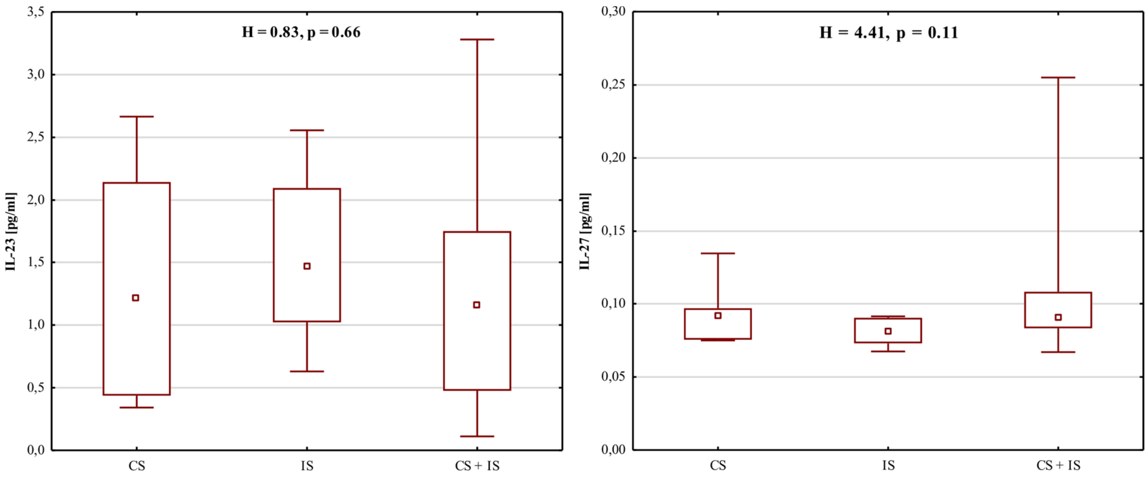

Figure S3. Association between IL-23 and IL-27 levels and type of treatment. CS, corticosteroids; IS, immunosuppressant medications; whiskers – min-max, inside box corresponds to the median, outside box boundaries correspond to 25% and 75%.
